# Spectroscopy learning: A machine learning method for study diatomic vibrational spectra including dissociation behavior

**DOI:** 10.1016/j.mex.2020.101127

**Published:** 2020-11-05

**Authors:** Shanshan Long, Jia Fu, Jun Jian, Zhixiang Fan, Qunchao Fan, Feng Xie, Yi Zhang, Jie Ma

**Affiliations:** a College of Science, Xihua University, Chengdu 610039, China; b Institute of Nuclear and New Energy Technology, Collaborative Innovation Center of Advanced Nuclear Energy Technology, Key Laboratory of Advanced Reactor Engineering and Safety of Ministry of Education, Tsinghua University, Beijing 100084, China; c College of Advanced Interdisciplinary Studies, National University of Defense Technology, Changsha 410073, China; d State Key Laboratory of Quantum Optics and Quantum Optics Devices, Laser Spectroscopy Laboratory, College of Physics and Electronics Engineering, Shanxi University, Taiyuan 030006, China

**Keywords:** Spectral prediction, Dissociation energy, Machine learning

## Abstract

Molecular spectroscopy plays an important role in the study of physical and chemical phenomena at the atomic level. However, it is difficult to acquire accurate vibrational spectra directly in theory and experiment, especially these vibrational levels near the dissociation energy. In our previous study (Variational Algebraic Method), dissociation energy and low energy level data are employed to predict the ro-vibrational spectra of some diatomic system. In this work, we did the following:

1) We expand the method to a more rigorous combined model-driven and data-driven machine learning approach (Spectroscopy Learning Method).

2) Extracting information from a wide range of existing data can be used in this work, such as heat capacity.

3) Reliable vibrational spectra and dissociation energy can be predicted by using heat capacity and the reliability of this method is verified by the ground states of CO and Br_2_ system.

## Graphical abstract

**Figure:**
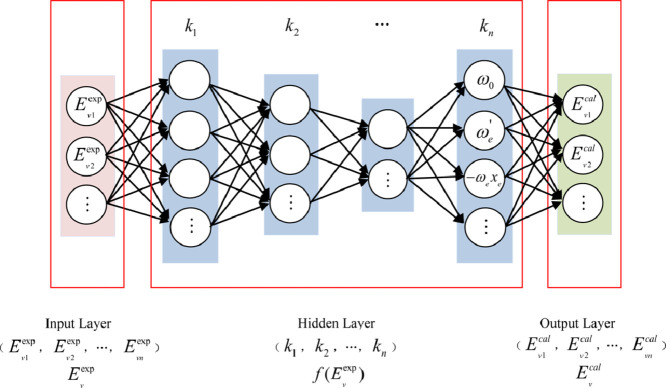

**Table:** 

**Specifications Table**	
Subject Area	Physics and Astronomy
More specific subject area	spectroscopy
Method name	Spectroscopy Learning Method (SLM)
Name and reference of original method	Variational Algebraic Method (VAM)
	Y. Zhang, W. Sun, J. Fu, Q. Fan, J. Ma, L. Xiao, S. Jia, H. Feng, H. Li, A Variational Algebraic Method used to study the full vibrational spectra and dissociation energies of some specific diatomic systems, Spectrochimica Acta Part A: Molecular and Biomolecular Spectroscopy. 117 (2014) 442–448.
Resource availability	

## Method details

For diatomic molecules, the spectral lines in the short-range region can be measured experimentally, while those lines lying in the mid-long-range region and the dissociation energy are difficult to measure. The spectroscopic parameters of the CO molecule are of great need in research of astrochemistry [1], and there remains interest in the Br_2_ molecule for the industrial applications [2, 3]. These two species are considered as promising candidates in the study of vibrational spectra. In recent years, machine learning has found a way to use data [4] to construct reliable higher-dimensional functions, which has shown good performance in solving problems of quantum mechanics and statistical mechanics [5], [6], [7]. In this paper, model-driven and data-driven methods are combined to predict the accurate vibrational spectra of diatomic molecules including dissociation energy by using limited experimental information, such as energy levels and heat capacity.

The method has three main parts.

1) Turning the spectroscopy problem to an optimizing machine learning problem. First, constructing a reasonable parametric model that can describe all the details of vibrational spectrum to solve under fitting problem (discussed in Section 1). Second, using machine learning strategy to focus on the over fitting problem (discussed in Section 2).
2) Introducing two main approach to solve under fitting problem. First, limiting the shape and size of parameter space (discussed in Section 3). Second, using testing data that including heterologous information to verify the predictive power of the selected model (discussed in Section 4).
3) Using greedy algorithm to search the optimal model in the parameter space (discussed in Section 5).

## 1 Model analysis from and beyond quantum models

According to Born–Oppenheimer approximation (BOA), the electronic Schrödinger equation of the diatomic molecules is given as

(1) $$\hat{H}\psi \left(r,R\right)=E\psi \left(r,R\right)$$

where $\hat{H}$ is the Hamiltonian of the *N*-electrons system, E is the electronic eigenvalue and ψ(r,R) stands for the total wave function of the system.

(2) $$\overset{\wedge }{H}=\overset{\wedge }{T}+\overset{\wedge }{V}=-\frac{\hbar^{2}}{2}\sum_{\alpha }\frac{1}{m_{\alpha }}\nabla_{\alpha }^{2}-\frac{\hbar^{2}}{2m_{e}}\sum_{i}\nabla_{i}^{2}+\sum_{\alpha }\sum_{\alpha >\beta }\frac{Z_{\alpha }Z_{\beta }e^{2}}{r_{\alpha \beta }}-\sum_{\alpha }\sum_{i}\frac{Z_{\alpha }e^{2}}{r_{i\alpha }}+\sum_{j}\sum_{i>j}\frac{e^{2}}{r_{ij}}$$

in which $Z_{\alpha }$ and $Z_{\beta }$ represent the charge number of nuclei α and β.

One is able to solve the radial nuclear Schrödinger equation for the vibrational energies and wave functions

(3) $$\left\{-\frac{\hbar^{2}}{2\mu }\frac{d^{2}}{dr^{2}}+V\left(r\right)+\frac{\left[J\left(J+1\right)-\Lambda^{2}\right]\hbar^{2}}{2\mu r^{2}}\right\}\varphi \left(r\right)=E_{vJ}\varphi \left(r\right)$$

where J, Λ and v represent the total angular momentum quantum number, the absolute value of the projection of the angular momentum of the electron orbit on the nucleus line (corresponding to the electronic state) and the vibrational quantum number respectively. $E_{vJ}$ is the rovibrational energy**.** And the potential energy function V(r) can be expanded to order 8 at the equilibrium position

(4) $$V\left(r\right)=\sum_{n=2}^{n_{\max}=8}\frac{1}{n!}f_{n}{\left(r-r_{e}\right)}^{n}=\sum_{n=2}^{n_{\max}=8}\frac{1}{n!}f_{n}x^{n}$$

where through the selection of the origin of coordinates, the constant term and the first-order term can be set as 0, $f_{n}=V^{\left(n\right)}\left(r_{e}\right)={\left(\frac{d^{n}V}{dr^{n}}\right)}_{r=r_{e}}$ is *n*-rank force constant.

Using the second order perturbation method to regroup the Hamiltonian in Eq. (3), the Hamiltonian can be written as

(5) $$\overset{\wedge }{H}=\overset{\wedge }{H_{0}}+\overset{\wedge }{H\prime }$$

where $\underset{}{\overset{\wedge }{H_{0}}}=-\frac{\hbar^{2}}{2\mu }\frac{d^{2}}{dx^{2}}+\frac{1}{2}f_{2}x^{2}$,$\overset{\wedge }{H\prime }=\frac{1}{6}f_{3}x^{3}+\ldots +\frac{1}{40320}f_{8}x^{8}+\frac{\left\{J\left(J+1\right)-\Lambda^{2}\right\}\hbar^{2}}{2\mu r^{2}}$, the final vibrational level is

(6) $$E_{vJ}=E_{v}^{\left(0\right)}+H\prime_{vv}+\sum_{m\neq v}\frac{{\left|H\prime_{vm}\right|}^{2}}{E_{v}^{\left(0\right)}-E_{m}^{\left(0\right)}}$$

The vibrational energies can be obtained with neglect of rotational part of the diatomic molecule

(7) $$E_{v}=\omega_{0}+\left(\omega_{e}+\omega_{e0}\right)\left(v+\frac{1}{2}\right)-\omega_{e}x_{e}{\left(v+\frac{1}{2}\right)}^{2}+\omega_{e}y_{e}{\left(v+\frac{1}{2}\right)}^{3}+\omega_{e}z_{e}{\left(v+\frac{1}{2}\right)}^{4}+\ldots$$

Where $\omega_{0}$,$\omega_{e0}$,$\omega_{e}x_{e}$,$\omega_{e}y_{e}$,$\omega_{e}z_{e}$…is the spectrum coefficients.

And the dissociation energy is only a function of the last three vibrational energies [8]

(8) $$D_{e}^{cal}≅E_{v_{\max}}+\frac{\Delta E_{v_{\max},v_{\max-1}}^{2}}{\Delta E_{v_{\max},v_{\max-2}}-\Delta E_{v_{\max},v_{\max-1}}}$$

According to Eq. (6) and Eq. (7), it can be found that the form of perturbation results is similar to the energy expansion formula of Herzberg [9] and the Dunham [10] formula derived by the method of WKB theory. Compare to Taylor's expansion

(9) $$f\left(x\right)=\frac{f(x_{0})}{0!}+\frac{f^{\prime }\left(x_{0}\right)}{1!}\left(x-x_{0}\right)+\frac{f^{{}^{\prime \prime }}\left(x_{0}\right)}{2!}{\left(x-x_{0}\right)}^{2}+\ldots +\frac{f^{\left(n\right)}\left(x_{0}\right)}{n!}{\left(x-x_{0}\right)}^{n}+R_{n}\left(x\right)$$

we can see that, E(v) can be expanded as series at $v=-\frac{1}{2}$. Any complex physical effect can be reacted by the coefficient of expansion, which is also known as the molecular constant [11] that are usually obtained by fitting the experimental data with the least square method. For the study of energy spectra of diatomic molecules, a large number of expansion terms are needed in the long-range region, so more spectral constants and polynomial terms are required. The problem is that, as the number of polynomial terms increases, the ability of fitting increases rapidly, which overwrite the physical effects from other factors, resulting in overfitting. However, if the relevant high-order constants and uncontrollable errors are abandoned, the degree of fitting will go down, so that the phenomenon of underfitting will appear. Therefore, the least square method is not applicable here [8], and we need to construct a new method to study the molecular vibration in the long-range region.

## 2 A data-driven approach base on machine learning

**Fig. 1:**
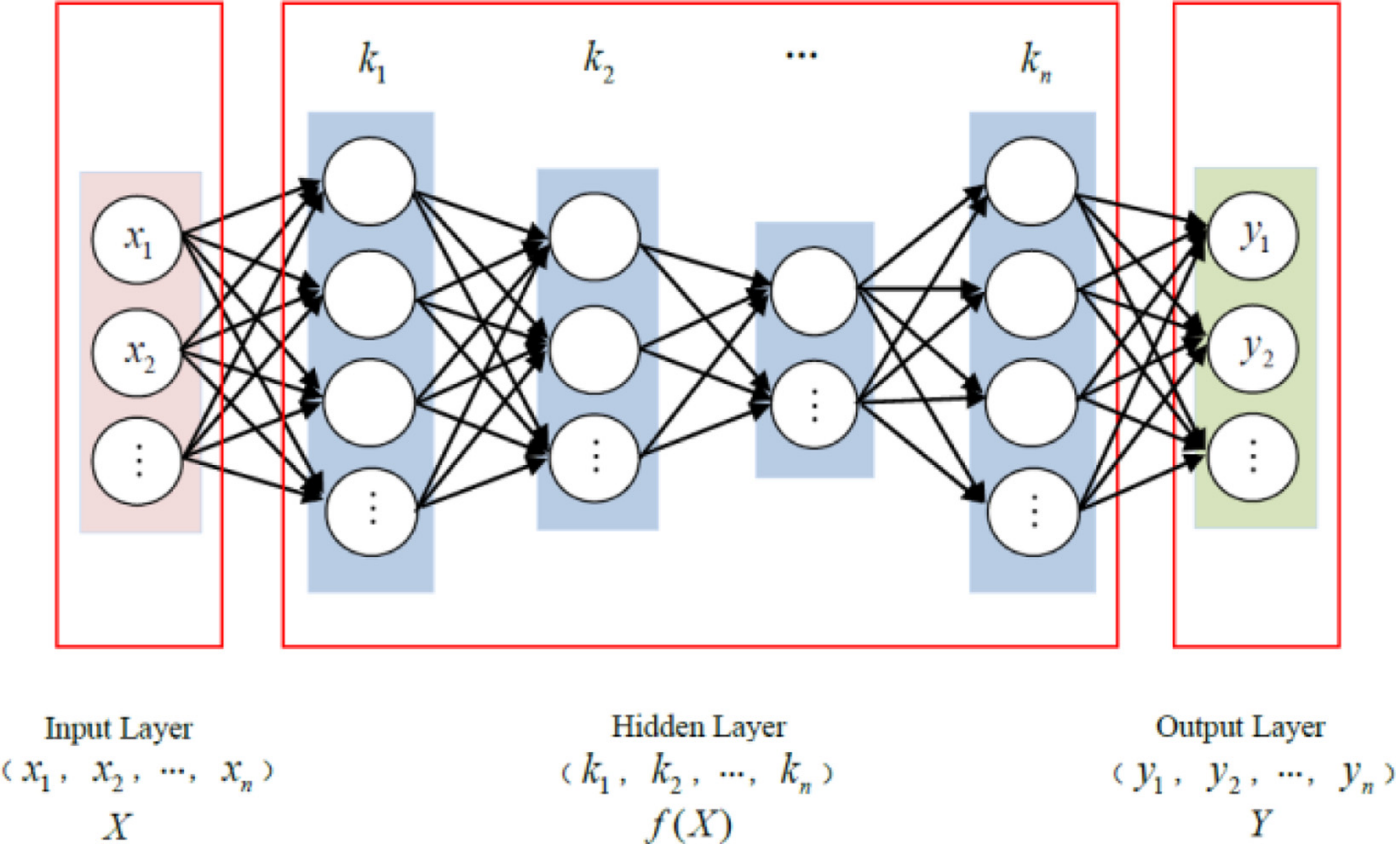
A typical Artificial neural network to build relationship between X and Y.

Machine learning method is to make use of the existing data (experience), get a certain model (parameter), so as to achieve the purpose of predicting unknown data. In the machine learning method, some very complex functions can be introduced, such as deep-neural-network (DNN), recurneural-network (RNN), convolutional neural-network (CNN), etc., which serves for general purpose like image classification. Their working principle is similar to the neural network shown in Fig. 1.

In general, machine learning means using data set carefully to determine the right mapping of X→Y. In order to solve the problem of underfitting, the number of parameters should be able to cover the established relationship when building the model. For the problem of overfitting, all data are divided into training set and test set. The data of the training set is used for learning to determine parameters, while the data of the test set is used to verify learning results. The overfitting problems can be further controlled by normalizing methods that introducing sufficient but limited parameter space to carry out effective model search [4].

Similarly, we found that Eq. (7) had the characteristics of fewer parameters and higher flexibility. And, the diatomic vibrational behavior relevant information (such as low-lying levels, dissociation energy and heat capacity) can be used as the training set and testing set. But, as mentioned above, overfitting is still the core challenge.

## 3 Limiting the shape and range of the parameter space

For diatomic molecular systems, the following matrix form can be obtained based on Eq. (7)

(10) AX=E

where

(11) $$A_{vk}={\left(v+\frac{1}{2}\right)}^{k},X=\left(\begin{array}{c} \omega_{0}\\ \omega_{e}^{\prime }\\ -\omega_{e}x_{e}\\ \vdots \end{array}\right),E=\left(\begin{array}{c} E_{v1}+\delta E_{v1}\\ E_{v2}+\delta E_{v2}\\ E_{v3}+\delta E_{v3}\\ \vdots \end{array}\right)$$

in which X is the spectral constant matrix, which is the parameter that we need to determine in the spectral learning process. The “real” energy levels E is break up to two parts: $E_{v1}$ stands for experimental measure value and δE [12] is a small variational term to offset any possible experimental error. According to Eq. (10), one can solve molecular constants X out:

(12) $$X=EA^{-1}$$

and the range of the parameter space (ΔX) is constrained by the experimental error:

(13) $$\Delta X=\Delta EA^{-1}$$

Occam's razor is used to further confine the shape of X. The simpler model (X) with enough expression are preferred. Usually, the dimension of X is set to 5 as a starting guess, and if 5 is not enough (Cannot represent the details of the data) then 6 will be used, and so on.

## 4 Preparing the data sets

Three different types of data are used to build the dataset.

1) The experimental vibrational energy $E_{v,\text{exp}}$. If the size of X is five, then five energy levels are enough to solve it out according to Eq. (10). The rest of the experimental levels can be used as validation set. For example, there are 42 experimental vibrational energies available for the ground electronic state of CO molecule. Assuming that the expansion order in the vibration energy term (see Eq. (7)) of this system is m(≥5), there are $C_{42}^{m}$ selections from the known 42 data to form the calculated subset for a certain m. So, we use levels as a part of training data.
2) Heat capacity [13] ($C_{mol}$) is introduced to enhance the training data set. The molar vibration heat capacity ($C_{mol}$) can be obtained experimentally and have a strong relation with the levels
  (14) $$C_{mol}^{cal}=\frac{N_{A}}{kT^{2}}\left(\langle E_{v}^{2}\rangle -{\langle E_{v}\rangle }^{2}\right)$$
3) Dissociation energy ($D_{e}$ in Eq. (8)). It is worth noting that this quantity may have very large uncertainty or lack of experimental data. From the probability point of view, if we can predict it correctly, it will greatly enhance the reliability of forecasts. So, we set it to test data.

## 5 Learning by optimizing

Now, parameter X is constrained by Eq. (13), and there are still many possibilities for its value. In learning steps, there are following objects to optimize:

(15) $$X^{*}=\underset{X}{\arg\min}∥E^{\exp}-AX∥$$

(16) $$X^{*}=\underset{X}{\arg\min}∥D_{e}^{\exp}-D_{e}^{cal}\left(X\right)∥$$

(17) $$X^{*}=\underset{X}{\arg\min}∥C_{mol}^{\exp}-C_{mol}^{cal}\left(X\right)∥$$

where $D_{e}^{cal}$and $C_{mol}^{cal}$ are determined by X, respectively from Eq. (8) and Eq. (14). The distance is defined as

(18) $$\bar{\Delta E}=\sqrt{\frac{1}{m}\sum_{v=0}^{m-1}{\left|E_{v,\exp}-E_{v,cal}\right|}^{2}}$$

for vibrational energies, and

(19) $$\bar{\Delta D_{e}}=\left|D_{e}^{cal}-D_{e}^{\exp}\right|$$

(20) $$\bar{\Delta C_{mol}}=\left|C_{mol}^{cal}-C_{mol}^{\exp}\right|$$

which represent dissociation energy and heat capacity respectively. Eq. (15) - Eq. (17) can be used to obtain $X^{*}$, however, in order to predict the vibrational spectra with neglect of experimental dissociation energy, we can only use Eq. (15) and Eq. (17)**.** That means taking $D_{e}$ as the unknown parameter and heat capacity is further introduced as an additional physical criterion to determine $D_{e}$.

In real calculation, we use the greedy algorithm to adjust $X^{*}$ parameter one by one, the calculation details are as follows:

1) For a certain $D_{e}$, five low-order parameters are used as initial attempt to determine the size of parameter X.
2) In the existing m experimental levels, one selects 5 experimental levels arbitrarily. Then 5 parameters in step 1) can be obtained according to Eq. (10). There can be totally $C_{m}^{5}$ different attempts, fortunately, you actually only need to try a few of them to find a satisfactory solution in practice.
3) Verify the parameters obtained in step 2) to see if they satisfy the criterion $\bar{\Delta E}<0.5cm^{-1}$ in Eq. (18) and $\bar{\Delta D_{e}}<10cm^{-1}$ in Eq. (19). What is noteworthy is that the criterion can also ensure that the final error given by the parameter solution found by different initial values in step 2) is very small.
4) If step 3) is met, the calculation ends. On the contrary, if the condition can't be met, keep the number of parameters to 5, a small variable item δE (usually 1 cm^−1^) is added to the first level, then, there are two new levels $\left(E_{v_{m}}-\delta E_{v_{m}},E_{v_{m}}+\delta E_{v_{m}}\right)$to solve out two new X, perform the validation in step 3) to see if the situation has improved. If not, the variable item becomes $\delta E^{\prime }$=$\frac{1}{2}\delta E$. If the conditions are still not met, the variable item will be halved again until reaching the upper limit in 10 times or achieve convergence (usually 0.001 cm^−1^). Next, adjust the next four energy levels in the same way.
5) If the conditions in step 3) are still not satisfied, increasing the number of parameters by 1(to 6 this time) and repeat steps 2) to 4) until step 3) is satisfied.
6) When step 5) is completed, the heat capacity can be calculated by Eq. (14) according to the spectrum. The heat capacity error curve of different dissociation energies can be drawn by changing the $D_{e}$ used in step 1), so that the optimal $D_{e}$ (the first inflection point) can be determined.
7) Compare $D_{e}$ with the experiment to see the quality of the prediction.

## 6 Method validation

We found the experimental dissociation energy values of the ground electronic state CO molecule over the years, as shown in Table 1.

**Table 1:** Dissociation energy of the ground electronic state for CO molecule [14].

$D_{e}$(cm^−1^)	Year	Method
55,821.120	1936	spectrum
70,976.136	1939	electron impact
75,815.428	1947	the theoretical calculation
81,461.247	1943	spectrum
89,615.437	1945	spectrum
90,679.1^1^	2014	spectrum

1 Newly added from [15].

**Fig. 2:**
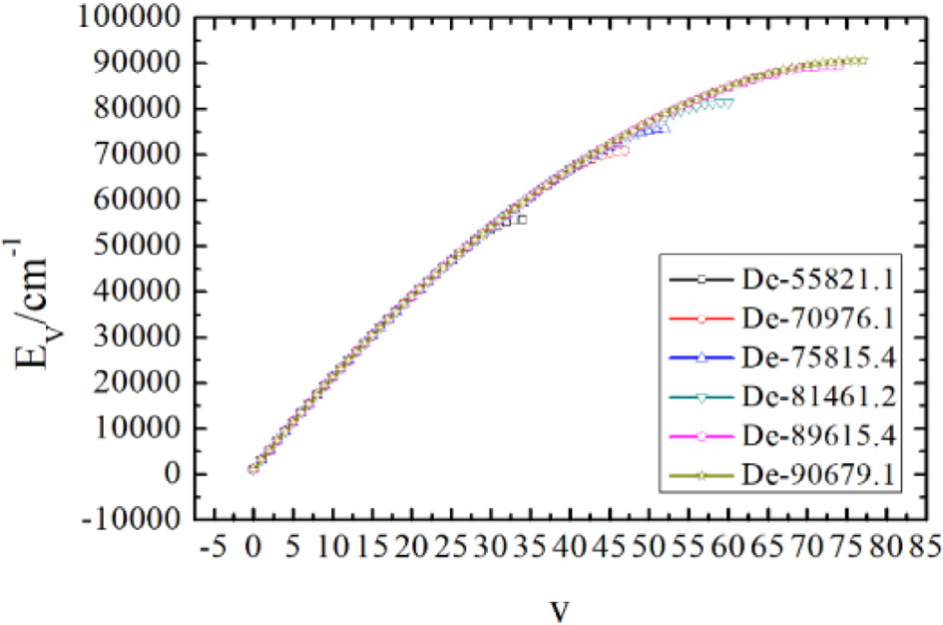
The full vibrational spectra corresponding to different dissociation energies for the ground electronic state of CO.

Hence the full vibrational spectra can be predicted using these different experimental dissociation energy values, as shown in Fig. 2, which were found to have great influence on the prediction of vibrational energies, especially those vibrational levels near the dissociation energy. And, better agreement can be found between the measurement and the present calculation using the dissociation energy $D_{e}=90679.1cm^{-1}$ [15], as shown in Table 2.

**Table 2:** Vibrational spectra of CO molecule in the ground electronic state.

v	$E_{v}^{\exp}$[16]	$E_{v}^{cal}$	v	$E_{v}^{cal}$
0	1081.701	1081.756	42	69,159.054
1	3225.042	3225.036	43	70,251.348
2	5341.833	5341.831	44	71,319.269
3	**7432.210**	7432.210	45	72,362.715
4	9496.241	9496.242	46	73,381.565
5	11,533.994	11,533.995	47	74,375.680
6	13,545.540	13,545.541	48	75,344.898
7	**15,530.954**	15,530.954	49	76,289.034
8	17,490.307	17,490.307	50	77,207.878
9	19,423.677	19,423.677	51	78,101.196
10	21,331.141	21,331.141	52	78,968.723
11	**23,212.778**	23,212.778	53	79,810.166
12	25,068.668	25,068.668	54	80,625.202
13	26,898.893	26,898.893	55	81,413.472
14	28,703.535	28,703.535	56	82,174.582
15	**30,482.679**	30,482.679	57	82,908.102
16	32,236.407	32,236.407	58	83,613.561
17	33,964.805	33,964.805	59	84,290.446
18	**35,667.957**	35,667.957	60	84,938.200
19	**37,345.949**	37,345.949	61	85,556.217
20	38,998.865	38,998.865	62	86,143.843
21	40,626.788	40,626.788	63	86,700.371
22	**42,229.802**	42,229.802	64	87,225.037
23	43,807.989	43,807.989	65	87,717.022
24	45,361.428	45,361.428	66	88,175.441
25	46,890.196	46,890.196	67	88,599.345
26	48,394.370	48,394.370	68	88,987.720
27	**49,874.020**	49,874.020	69	89,339.474
28	51,329.216	51,329.216	70	89,653.443
29	52,760.022	52,760.022	71	89,928.381
30	54,166.498	54,166.498	72	90,162.961
31	**55,548.698**	55,548.698	73	90,355.764
32	56,906.672	56,906.672	74	90,505.279
33	58,240.461	58,240.460	75	90,609.901
34	59,550.101	59,550.099	76	90,667.917
35	60,835.619	60,835.616	77	90,677.513
36	62,097.034	62,097.029		
37	63,334.355	63,334.347		
38	64,547.581	64,547.568		
39	65,736.698	65,736.681		
40	66,901.681	66,901.660		
41	68,042.490	68,042.469		
$D_{e}^{\exp}$	90,679.1 [15]		$D_{e}^{cal}$	90,679.099

**Fig. 3:**
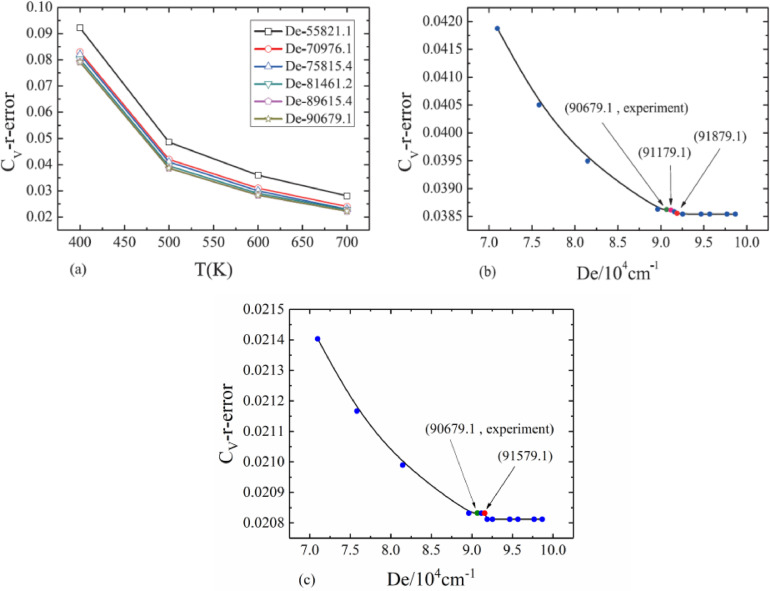
The relative errors between the theoretical and experimental vibrational molar heat capacity based on different dissociation energy for the ground electronic state of CO (a) under *T* = 400 K, 500 K, 600 K, 700 K; (b) under *T* = 500 K [17]; (c) under *T* = 1200 K [17].

We take the dissociation energy as an unknown quantity and use the relative error between the calculated ($C_{mol}^{cal}$) and experimental heat capacity ($C_{mol}^{\exp}$) as the standard to search for the dissociation energy which can best meet our requirements. As shown in Fig. 3(a), more accurate the dissociation energy is, more reliable the calculated vibrational energies will be. Again, the best choice for the dissociation energy is still $D_{e}=90679.1cm^{-1}$ [15].

The dependence of dissociation energy on heat capacity provides a way to obtain dissociation energy and makes it a good criterion to verify the reliability of this method. The results show that, as shown in Fig. 3(b)(*T* = 500 K), with the increase of dissociation energy, the relative error of heat capacity decreases gradually, and the corresponding dissociation energy at the first inflection point is close to the latest experimental value (90,679.1cm^−1^). Since the heat capacity also have its uncertainty, we can ignore the details of the change after the first turning point($D_{e}>91179.1cm^{-1}$), so that the 91,179.1 cm^−1^ we found can be used as an estimate of the absolute error within 500 cm^−1^ (5.5 ‰), which is better than the second best dissociation energy value in Table 1. As shown in Fig. 3(c), and we also set a second temperature(*T* = 1200 K) to find the right dissociation energy($D_{e}=91579.1cm^{-1}$),which is a little bit worse than what we just did.

**Fig. 4:**
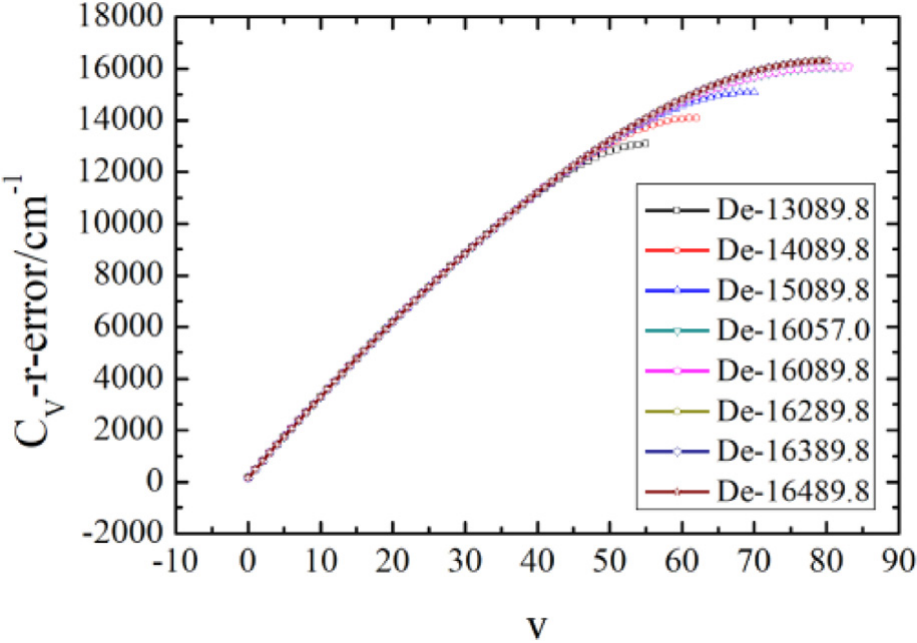
The full vibrational spectra corresponding to different dissociation energies for the ground electronic state of Br_2_.

**Fig. 5:**
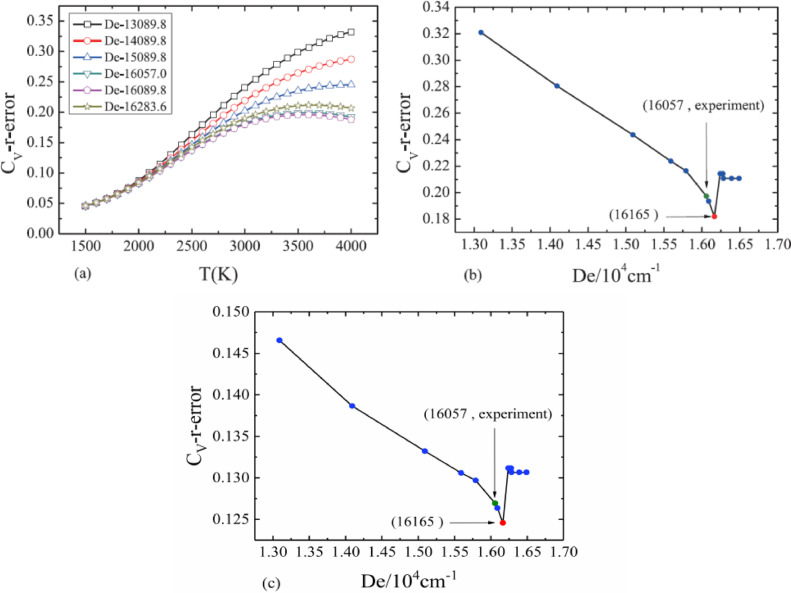
The relative errors between the theoretical and experimental vibrational molar heat capacity based on different dissociation energy for the ground electronic state of Br_2_ (a) under *T* = 1500 K — 4000 K; (b) under *T* = 3800 K [17];(c) under *T* = 2400 K [17].

In order to further verify the effectiveness and practicability of this method, similar analysis for the ground electronic state of Br_2_ is carried out. Several candidate points were selected near the experimental dissociation energy (16,057 cm^−1^ [18]), which can yield a group of vibrational energies for each case, as shown in Fig. 4. The dissociation energy can be determined the same as those in CO molecule and is given as 16,165 cm^−1^ with the help of vibrational molar heat capacity as the requirement. In addition, the result shown in Fig. 5(c) at the second temperature(*T* = 2400 K) is consistent with that just shown in Fig. 5(b)(*T* = 3800 K), and the dissociation energy also given as 16,165 cm^−1^.

## Declaration of Competing Interests

The Authors confirm that there are no conflicts of interest.
